# Hepatitis B prevention: Can we learn from the response to HIV/AIDS?

**DOI:** 10.1371/journal.pmed.1003109

**Published:** 2020-04-21

**Authors:** Mirjam E. E. Kretzschmar, Marianne A. B. van der Sande

**Affiliations:** 1 Julius Center for Health Sciences and Primary Care, University Medical Center Utrecht, Utrecht University, Utrecht, The Netherlands; 2 Centre for Infectious Disease Control, National Institute of Public Health and the Environment (RIVM), Bilthoven, The Netherlands; 3 Department Public Health, Institute of Tropical Medicine, Antwerp, Belgium

## Abstract

Mirjam Kretzschmar and Marianne van der Sande discuss the accompanying research study by Anna McNaughton and colleagues on strategies to reduce the burden of hepatitis B in African countries.

Following the successful introduction of infant hepatitis B vaccination in routine vaccination programmes, the prevalence of chronic hepatitis B infection has declined [1]. Nevertheless, prevalence still remains above 10% in many regions of Africa, as demonstrated by the accompanying study by Anna McNaughton and colleagues in *PLOS Medicine* [2]. Drawing together evidence from various cohort studies and seroepidemiological datasets, the authors provide a comprehensive map of the distribution of chronic hepatitis B in adults in Africa. They also analysed distribution of the prevalence of anti–hepatitis B core antigen (HBc) as a serological marker of previous exposure to hepatitis B virus (HBV) and correlate of protection. Knowing the prevalence of active infection and previous exposure, the proportion of the adult population still susceptible to infection can be inferred, and the impacts of interventions can be estimated.

McNaughton and colleagues used a mathematical model [3] to assess impact of test-and-treat strategies. These are inspired by the strategy of treatment as prevention for HIV, which has become one of the main pillars for HIV prevention in resource-limited settings [4]. Simulations show that including diagnosis and treatment of chronic hepatitis B infections in programmes could reduce the prevalence of chronic infections by 62% (95% CI 57%–68%) within a time period of 50 years [2].

## Complex dynamics of hepatitis B infections

McNaughton and colleagues are to be commended for their approach of including regional data on anti-HBc prevalence. This enables a better understanding of the transmission dynamics of the virus, which may explain why vaccination has been more effective in some populations than in others. Dynamics of HBV are complex because transmission may occur along several routes, including perinatal transmission from mother to child, horizontal transmission within households (especially among young children), bloodborne transmission, and sexual transmission. The average age of infection differs by route of transmission, and this age determines the rate of developing chronic infection versus recovery with subsequent immunity. The age-dependent rate of developing chronic infection may facilitate the occurrence of two alternative endemic situations, namely, one driven by acquisition of the virus in childhood and high proportions of chronic infections and another driven by sexual transmission and low rates of chronicity [5]. Populations may transition from one to the other, whereby large shifts in prevalence of chronic infection may occur, thereby changing the relationship between hepatitis B surface antigen (HBsAg) and anti-HBc prevalence. In Africa, most chronic infections to date are the result of incident infections in early life, when the risk of chronicity is very high, while nearly all new infections in adulthood will resolve and lead to immunity (i.e., anti-HBc positivity). Reducing transmission risks among African adults will have most impact on prevalence through reducing risk of early life infections.

As chronic hepatitis B, like HIV infection, is a lifelong condition, changes in hepatitis B seroprevalence in a population will be slow. Even if infant vaccination rates are high, it will take at least one generation before prevalence of exposure drops significantly. The prevalence of chronic infection that we observe now has been shaped by transmission dynamics in past decades and by the impact of infant vaccination since the 1990s. Depending on how fast universal infant vaccination was implemented, the impact differs between regions. The WHO Global Hepatitis Report 2017 showed that, in the African region, vaccination coverage only started to increase to substantial levels at the beginning of the 21st century, reaching 75% around 2008 [6]. The coverage of the birth dose, i.e., vaccination given in the first 24 hours to reduce the risk of mother-to-child and early life transmission, has remained very low in the African region, keeping the transmission pattern of early infection with high chronicity in place. A study in The Gambia [7] showed that, while 93.1% of children were vaccinated by 6 months of age, only 1.1% of children were vaccinated at birth and 5.4% by day 7, leaving a considerable period during which chronically infected mothers and other close contacts can infect infants. Such delays in providing vaccine-induced protection to infants in environments with high exposure are likely to be a key factor in why prevalence has not decreased more rapidly since highly effective vaccination became available.

## Lessons learned from efforts to control HIV

Clearly, if elimination of viral hepatitis is a goal to be reached within the lifetime of children born today, we need to complement vaccination campaigns with additional interventions. It is logical to ask what we can learn from the global fight against HIV [8]. HIV prevention has included universal test-and-treat strategies since the publication of the Joint United Nations Programme on HIV/AIDS (UNAIDS) 90-90-90 document [9], aiming to reach HIV elimination by 2030. The rationale of test-and-treat strategies is to combine the benefit of treatment for the individual with the public health benefit of preventing onward transmission. In HIV, the rollout of antiretroviral therapy has led to reductions in incidence of new infections [10–12]. A similar effect can be expected from treating hepatitis B infections, whereby—comparable to HIV—test-and-treat strategies should contribute to achieving HBV elimination.

Nevertheless, there are practical challenges in implementing test-and-treat strategies for hepatitis B. First, there is a need for rapid reliable diagnostic tests, affordable for the target populations, with robust transport and storage conditions. So far, no available tests have been prequalified by WHO or approved by the Food and Drug Administration (FDA) [13]. Next, adherence to lifelong therapy calls for careful support to patients and attention to their social environments to address potential barriers—including financial, logistical, and psychological barriers—or stigma, as well as to provide access to specialized medical follow-up to manage potential side effects. Here, lessons learned from supporting adherence to HIV treatment will be helpful. Finally, a strategy needs to be developed for whom to test in a manner that is sustainable and acceptable in resource-constrained environments. Targeting women attending antenatal care would be a logical start. This could build on experience with HIV, include combination screening for HBV and HIV, and promote timely vaccination of infants. Making HBV test-and-treat strategies part of a comprehensive strategy to strengthen pre-, peri-, and postnatal care for pregnant women and their infants would not only support the drive for HBV elimination [14] but also contribute to strengthening maternal and child health and achieving the health component of the Sustainable Development Goals in the wider context.

## Abbreviations

FDA: Food and Drug Administration
HBc: hepatitis B core antigen
HBsAg: hepatitis B surface antigen
HBV: hepatitis B virus
UNAIDS: Joint United Nations Programme on HIV/AIDS
